# Spinal Muscle Thickness and Activation during Abdominal Hollowing and Bracing in CrossFit^®^ Athletes

**DOI:** 10.3390/sports11080159

**Published:** 2023-08-18

**Authors:** Ioannis Tsartsapakis, Georgia-Andriana Pantazi, Agapi Konstantinidou, Aglaia Zafeiroudi, Eleftherios Kellis

**Affiliations:** 1 Laboratory of Neuromechanics, Department of Physical Education and Sport Sciences at Serres, Aristotle University of Thessaloniki, 62100 Serres, Greece; ioantsar@phed-sr.auth.gr (I.T.);; 2 Department Physical Education & Sport Science, University of Thessaly, 42100 Trikala, Greece

**Keywords:** abdominal hollowing, abdominal bracing, core muscles activation, ultrasound, EMG, CrossFit^®^

## Abstract

Exercises that improve muscle activation are essential for maintaining spinal stability and preventing low back pain. The purpose of this study was to compare the effects of abdominal hollowing and bracing on the activation of the core muscles in CrossFit^®^ participants using ultrasound and electromyography (EMG). Twenty-four healthy adults aged 21 to 42 years old with at least two years of CrossFit^®^ experience performed three core stability exercises (plank, side plank, bridge) with abdominal hollowing and bracing. We measured the ultrasound relative thickness of the local core muscles (transversus abdominis, internal oblique, and lumbar multifidus), and the EMG percentage of maximal voluntary contraction (MVC) of the global core muscles (rectus abdominis, external oblique, and iliocostalis lumborum). Analysis of variance tests showed that the relative thickness of the local core muscles was greater (*p* = 0.016) during hollowing (range from 26.8 ± 5.33 to 88.4 ± 11.9% of rest) than bracing (range from 15.9 ± 3.54 to 61.2 ± 15.9% of rest), while the EMG of the global muscles was greater (*p* = 0.001) in bracing (range from 24.4 ± 7.30 to 72.5 ± 9.17% of MVC) than hollowing (range from 16.4 ± 3.70 to 56.6 ± 7.65% of MVC). These results indicate that the recruitment of spinal muscles during popular exercises is achieved with both hollowing and bracing. Nevertheless, it appears that hollowing tends to recruit more of the local muscles, whilst bracing recruits more of the global muscles. The grading of the exercises varied between muscles and varied between maneuvers, especially for the surface abdominals and lumbar muscles. CrossFit^®^ practitioners can choose to use either hollowing or bracing to activate their core muscles more selectively or more appropriately, depending on the goal and purpose of the exercise.

## 1. Introduction

CrossFit^®^ is a fitness program that combines gymnastics, strength training, diet, and various exercises that use aerobic and anaerobic energy systems to enhance strength and endurance in different physical domains [1,2,3]. Such programs are recommended for both genders [4] and for athletes and non-athletes who want to improve their cardiovascular/respiratory endurance, stamina, flexibility, power, speed, coordination, agility, balance, and accuracy. However, CrossFit^®^ also has some challenges and risks for its participants, such as a higher rate of injury [1,5] and overtraining syndrome [1,2]. Shoulder, lumbar spine, and knee injuries are quite common among CrossFit^®^ practitioners [5,6]. Therefore, it is important to know the factors that can affect the safety and effectiveness of CrossFit^®^ exercises and how they can be optimized to achieve better performance and health results.

CrossFit^®^ exercises involve different kinds of movements and loads that require spinal stability and performance [1,2,3]. Core muscles are the muscles of the abdomen, back, pelvis, and hips that support the spine and prevent excessive movements that can cause injury [7]. The term “core” refers to muscles that tend to stabilize the spinal cord, whilst “global” muscles are considered those muscles that tend to produce torque relative to the spine axis of rotation [8,9,10]. Even though the use of such terms is debated [11,12], the quantification of the activation of the spinal muscles is important for understanding the effectiveness of CrossFit^®^ exercises for improving trunk muscle function.

There are different ways of activating the spinal muscles, such as abdominal hollowing and bracing. Abdominal hollowing refers to the maneuver where the participant pulls the navel towards the spine, while abdominal bracing means contracting the core and surrounding muscles. Nevertheless, there is some disagreement about which way is more effective in stabilizing the lumbar spine and improving performance in different types of exercise [12,13]. Hence, exercise specialists and practitioners are often not certain which type of maneuver is more optimal for enhancing trunk stability and mobility.

A comparison of hollowing and bracing while performing various exercises, as well as between exercises and muscles, is often made using electromyography (EMG) [14,15,16,17,18] and ultrasound (US) [19,20,21,22]. US imaging can reliably evaluate changes in the size of key local core muscles, such as the transversus abdominis (TrA), the deeper part of the lumbar multifidus (LM), and the internal oblique (IO) [23]. This technique provides an index on muscle activation via the measurement of muscle thickness at rest and during contraction [24]. Surface EMG can provide measurements on the activation of the superficial (global) muscles, such as the surface part of the iliocostalis lumborum (IL), the external oblique (EO), and the rectus abdominis (RA) [19,25]. US and EMG can be used together to provide complementary information about the neuromuscular system [26,27].

Many studies [14,15,16] found that hollowing shows greater TrA/IO abdominal surface or intramuscular EMG activation than bracing, while no differences in RA activation were detected [14,15]. Some studies reported that bracing causes greater EMG in the EO [15,18] and IO [18] than when hollowing, while one study reported greater TrA activation during bracing than hollowing [18]. Using both intramuscular and surface EMG measurements, a recent study [17] reported that abdominal bracing caused a greater RA activation and a lower IO and EO activation than hollowing, whilst the TrA activation did not differ between the two maneuvers. Collectively, these EMG findings indicate that bracing tends to cause greater activation of the global muscles than hollowing, but there are conflicting findings as to the differences in deeper muscle activation between the two maneuver types, while it becomes apparent that these differences may also vary between exercises and muscles.

A few studies have compared the US thickness between hollowing and bracing techniques [19,20,21,22]. Moghadan et al. [19] found that while performing the bridge exercise there was a greater TrA thickness change during abdominal bracing compared to hollowing from various exercise positions. In contrast, others reported an almost double-fold change in the TrA thickness during hollowing compared to bracing from the supine [20,21], sitting [21] and standing [22] position. Furthermore, some investigators reported greater IO and EO thickness during hollowing than bracing [21] whilst others reported the opposite [22]. Using magnetic resonance imaging after exercise, Koh et al. [28] found that the bracing maneuver increased the cross-sectional area of the rectus abdominis and the oblique abdominals, while the hollowing maneuver selectively activated the TrA. From the above studies, it appears that even though each maneuver type changes the thickness of the deep abdominals relative to the standard procedure, it is unclear which maneuver type causes a greater increase in the deep abdominal wall muscle thickness.

Understanding the level of spinal muscle activation is crucial for appreciating the safety and usefulness of CrossFit^®^ programs. Nevertheless, to the best of our knowledge, no previous study has examined the activity of surface and deep spinal muscles during abdominal hollowing and bracing during popular exercises using simultaneous US thickness and EMG measurements. Further, the capacity to recruit the abdominal wall muscles may differ depending on the population type [24,29], as well as the type of exercise. For this reason, investigating the influence of bracing and hollowing on spinal muscle recruitment in CrossFit^®^ trainees is worthwhile. Therefore, the purpose of this study was to compare the effects of abdominal hollowing and bracing on US and EMG indicators of core muscles in CrossFit^®^ participants. The main research question was: How do local muscles, such as the TrA, IO, and LM, measured by US differ and how does activation of global muscles, the RA, EO, and IL, differ between abdominal hollowing and bracing? A secondary question was whether differences in the muscle recruitment between various exercises depend on whether participants perform the hollowing or bracing maneuver.

## 2. Materials and Methods

### 2.1. Study Design

**Figure 1:**
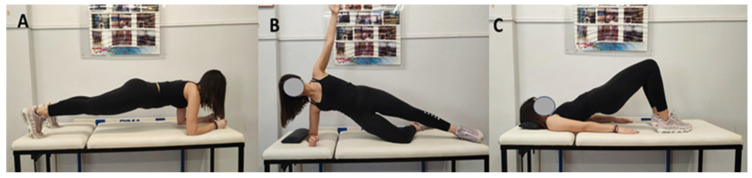
Illustration of the examined exercises: (**A**) plank, (**B**) side plank, (**C**) bridge. These photos are presented with the consent of the subject.

We conducted power analysis using the G*Power software (v. 3.1.9.4, Heinrich-Heine-Universität Düsseldorf, Düsseldorf, Germany) to determine the required sample size for our study. Based on previous research findings [30,31], we estimated the effect size at 0.35. We set the level of significance at α = 0.05. The G*Power analysis yielded the following results: critical F-value = 3.2, numerator degrees of freedom = 2.0, denominator degrees of freedom = 44.00, and a total required sample size of 24 participants. The calculated statistical power was 96%, indicating a high likelihood of detecting significant effects if they exist (Figure 1). We used a repeated measures protocol to examine local and global core muscle changes between rest (US), maximum volume activation (EMG), and the three core stability exercises with the two activation maneuvers. We interviewed all the participants in the daytime in September 2022 and excluded those who did not meet the inclusion criteria. Although physiological health and performance indicators were outside the scope of this study, we provided dietary and hydration guidelines. After the initial data collection, which included body measurements, we informed the subjects of the exercise protocol and the correct way to perform the two maneuvers for core muscle activation. Following a short period of practical familiarization with the exercises and maneuvers of activation, the subjects performed each of the exercises three times for the collection of the survey data.

### 2.2. Sample

This cross-sectional study included 24 healthy adults (9 females), with a mean of 29.5 ± 7.83 years (between 21 to 42 years old), with a minimum of 2 years (M = 4.35 ± 2.66) experience with CrossFit^®^ training. The participants were recreationally active and were instructed not to exercise strenuously for at least three days prior to testing, and not to drink alcohol for at least two days prior to testing. To be included in the study, the subjects had to be healthy, 18 years or older, and have no discomfort or injury in the sciatic region of the spine. The exclusion criteria from the study included those with spinal, shoulder, and knee injuries; those who were novices (less than two years) to CrossFit^®^ training; those who could not perform the two maneuvers (hollowing, bracing) to activate the abdominal and dorsal muscles; and the intake of medications that potentially affect reaction times. Of the 44 people who volunteered to take part in the survey, only 24 of them met the criteria for inclusion in the study.

The participant characteristics are presented in Table 1. The study followed the latest version of the Declaration of Helsinki and the ethical guidelines from the Aristotle University of Thessaloniki. After being informed about the purpose of the study, all the participants gave their written consent.

### 2.3. Instruments

We used a computerized ultrasonography (US) system (Aloka ProSound SSD 3500 SV, Aloka Co. Ltd., Tokyo, Japan), with a 6 cm transducer head and 13 MHz frequency to measure the deep core/abdominal muscles: TrA, IO, and LM.

Similarly, the EMG of the surface muscles (RA, EO, and IL) were measured using bipolar bar surface electrodes (inter-electrode distance 1 cm; TSD 150 B, Biopac Systems Inc., Goleta, CA, USA) and shielded surface electrode lead assemblies (model SS2, Biopac Systems Inc., Goleta, CA, USA) connected to an amplifier/transmitter, sampling at 1024 Hz (model TEL100 M, Biopac Systems Inc., Goleta, CA, USA; common mode rejection > 110 db at 50/60 Hz; bandwidth = 10–500 Hz; gain 1000) using the Acknowledge (version 3.9.1, Biopac Systems) software. The hip and knee joint positions during each exercise were measured with a standard analogue goniometer to an accuracy of ±1° (model 01135, Lafayette Instrument Company, Lafayette, IN, USA).

### 2.4. Exercise Protocol

The experiment was performed in a laboratory setting and during the same time of day for all the participants. We set the total duration of the procedure to 55 min for each participant, in order to avoid fatigue in the trunk area. We first instructed the participants on how to perform the abdominal bracing and abdominal hollowing muscle activation methods and had them practice each exercise several times. After familiarization, we collected the main measurements.

First, with the participants in the supine position, we measured the relaxed muscle thickness of the TrA and IO by US. The relaxed muscle thickness of the LM was measured by US during the prone position. We also measured the maximum volume activation of the RA, EO, and IL using EMG.

The participants performed three trunk isometric exercises with two ways of engaging their core muscles: bracing and hollowing. Each exercise lasted 10 s, during which the thickness (US) or muscle activation (EMG) was measured. The participants performed the three exercises as follows. First, they did the plank exercise, in which they laid on the floor with their forearms and toes raising their body off the floor, whilst keeping their body straight and holding the position for 10 s (Figure 1A). Next, they did the side plank exercise, in which they laid on one side with their elbow and knee raising their body off the floor, lifting their hips and holding the position for 10 s (Figure 1B). Finally, they did the bridge exercise, in which they laid on their back with their knees bent, pushed their hips up and held the position for 10 s. Figure 1 shows the three exercises. Detailed instructions for the participants can be found in Supplementary Materials, Supplementary File S1.

### 2.5. US Measurements

To measure the TrA and the IO, we placed the US head 2.5 cm above the iliac crest and along the axillary line [24]. We oriented the probe in the transverse plane, perpendicular to the muscle fibers, and 2 cm to the left of the middle of the US image when relaxed. The TrA is the deepest layer of the abdominal muscles, followed by the IO and then the EO. We measured the TrA and IO thickness as the distance from the superior to the inferior fascia of the muscle.

To measure the LM, we placed the US head on the lower back, parallel to the spine, at the level of the L3-L4, L4-L5, or L5-S1 intervertebral spaces. We oriented the probe in the longitudinal plane, parallel to the muscle fibers. The deep part of LM is located deeper than the erector spinae muscle. We measured the LM thickness as the distance from the superior to the inferior fascia of the muscle. In addition to the absolute thickness (measured in mm), we expressed the relative thickness ratio during each exercise as a percentage of the resting thickness for each muscle and each exercise: (contraction- rest)/rest × 100 [24].

### 2.6. EMG Measurements

The electrode positions were first identified on the skin and then cleaned with alcohol wipes to remove dead skin and reduce impedance. The electrode positions were chosen in accordance with the SENIAM project guidelines [32]. For the IL, the electrodes were placed about 2 cm medial from the line from the posterior spina iliaca to the lowest point of the lower rib, at the level of L2. For the RA, the electrodes were centered on the muscle belly, vertically near the midpoint between the xiphoid process and the pubic symphysis and 3 cm lateral from the midline. For the EO, the electrodes were positioned obliquely about 45° above the anterior superior iliac spine, at the level of the umbilicus. For the IO, the electrodes were positioned in the horizontal direction, about 2 cm anteromedial to the anterior superior iliac spine [33]. A common ground electrode was also placed on the wrist or ankle of the individual.

To test the maximal voluntary contraction of the RA, the participants laid on their backs with their knees bent at about 90 degrees. They tried to flex their trunks against a restraint strap on their chests as hard as they could. To test the EO, they followed the same protocol but bent their trunk sideways instead. To test the IL, the participants laid in a prone position with their legs straight and their arms by their sides. They were instructed to lift their upper back against a restraint strap as hard as they could.

The raw EMG signal was filtered using a band pass filter (low 15 Hz and high 450 Hz, full wave rectified) and the root mean square (RMS) (with a step of 50 ms) was recorded. The maximum RMS value, which was produced during the isometric MVC of the RA, EO, and IO, was taken as a reference measurement. Subsequently, the mean RMS, which was recorded during the 10 s holding phase of each exercise, was normalized to the MVC value. 

### 2.7. Statistical Analysis

All the statistical analyses were conducted using IBM SPSS Statistics ver. 28.0 (IBM Co., Armonk, NY, USA). The normal distribution of the collected data was verified by using the Kolmogorov–Smirnov test. We used three-way (muscles × exercises x maneuver) repeated measures analysis of variance (ANOVA) tests to analyze the difference in the muscle thickness or EMG measurements between the conditions. Post-hoc Bonferroni tests were applied to investigate the pairwise differences in the exercises, muscles, and maneuver methods. Partial eta squared (η*_p_*^2^) values were also calculated to indicate the effect size. The Greenhouse–Geisser correction was used when the assumption of the sphericity was violated. The level of significance was set at *p* < 0.05.

## 3. Results

### 3.1. Ultrasound Relative Thickness

The mean US relative muscle thickness descriptive scores for each exercise and method are presented in Table 2: a three-way repeated measures ANOVA revealed a significant three-way (muscle by exercise by method) interaction (*F*_(4.92)_ = 4.020, *p* = 0.016, η*_p_*^2^ = 0.149) in the relative thickness values. The Bonferroni post-hoc tests showed that the TrA (*p* < 0.01), IO (*p* < 0.001), and LM (*p* < 0.01) relative thicknesses were statistically significantly greater in all three exercises using hollowing compared to the thicknesses when performing the bracing protocol.

Regarding the differences between the muscles, the TrA and IO relative thicknesses were statistically significantly greater compared to the LM (*p* < 0.01) relative thickness in all three exercises. Furthermore, the TrA relative thickness was statistically significantly greater compared to the LM thickness in all (*p* < 0.01) but one exercise condition (bridge, bracing).

**Figure 2:**
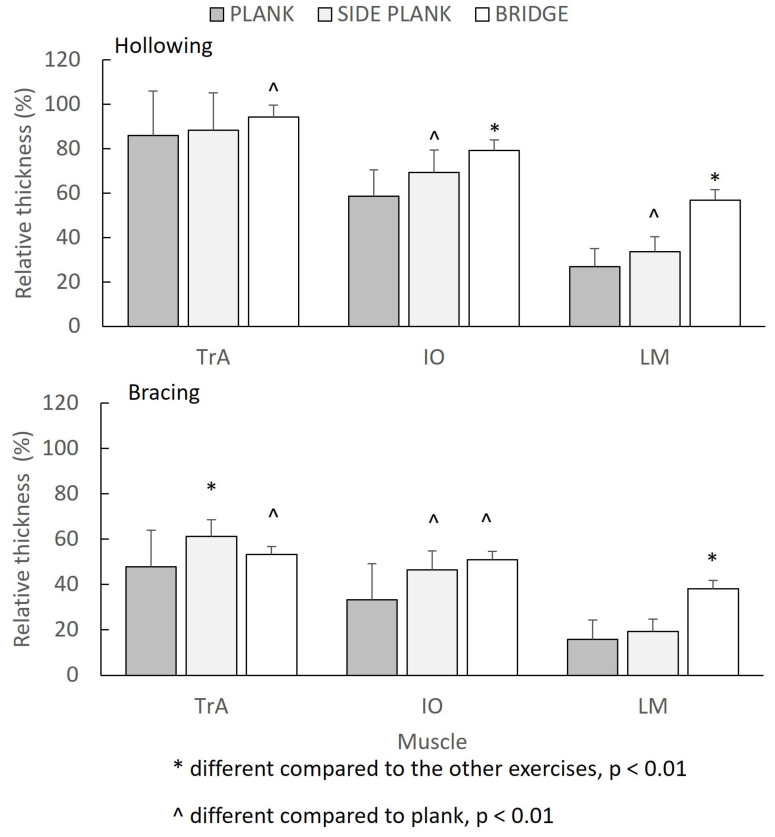
Relative muscle thickness of the transversus abdominis (TrA), internal oblique (IO), and lumbar multifidus (LM) for each exercise and maneuver.

Figure 2 illustrates the influence of the exercise and type of maneuver for each muscle. The exercises that showed the greatest relative thickness differed between the muscles and depended on whether the individuals used the hollowing or bracing technique. When hollowing was performed, the TrA thickness was greater during the bridge exercise compared to the plank exercise (*p* < 0.01), while the TrA thickness did not differ between the side plank and the plank exercise or between the side plank and the bridge (*p* > 0.05). In contrast, when exercises were performed with simultaneous bracing, the TrA was greater during the side plank compared to the plank and bridge exercise (*p* < 0.01) and the side plank also showed greater relative thickness compared to the plank (*p* < 0.01).

The IO relative thickness was significantly different between the three exercises during the hollowing condition, in the following order: bridge > side plank > plank (*p* < 0.01). When using the bracing technique, the IO relative thickness was lower during the plank exercise (*p* < 0.01), but it was no different between the bridge and side plank (*p* > 0.05). Finally, the LM relative thickness was greater during the bridge compared to the plank and side plank exercises (*p* < 0.01), while the side plank exercise showed greater LM relative thickness than the plank exercise only during the hollowing condition (*p* < 0.01).

### 3.2. EMG Muscles Activation

The mean EMG muscle normalized activation for each exercise and method is shown in Table 3. The ANOVA showed a significant three-way (muscle–exercise–method) interaction (*F*_(4.92)_ = 20.052, *p* = 0.0001, η*_p_*^2^ = 0.466) for normalized EMG values.

The Bonferroni post-hoc comparisons showed that the RA, EO, and IL EMG during bracing were greater than hollowing in all three exercises (*p* < 0.001, η*_p_*^2^ = 0.970). The differences between the muscles varied between the exercises. In the plank exercise, the RA normalized EMG was greater than the EO and IL activation (*p* < 0.01), whilst the EO was statistically significantly more activated than the IL (*p* < 0.01). For the side plank exercise, the greatest EMG activity was displayed by the EO (*p* < 0.01), whilst the IL had a significantly greater normalized EMG than the RA activation (*p* < 0.01). Finally, for the bridge exercise, the IL normalized EMG was statistically significantly greater than the RA and EO values (*p* < 0.01), whilst the EO EMG was greater than the RA (*p* < 0.01).

**Figure 3:**
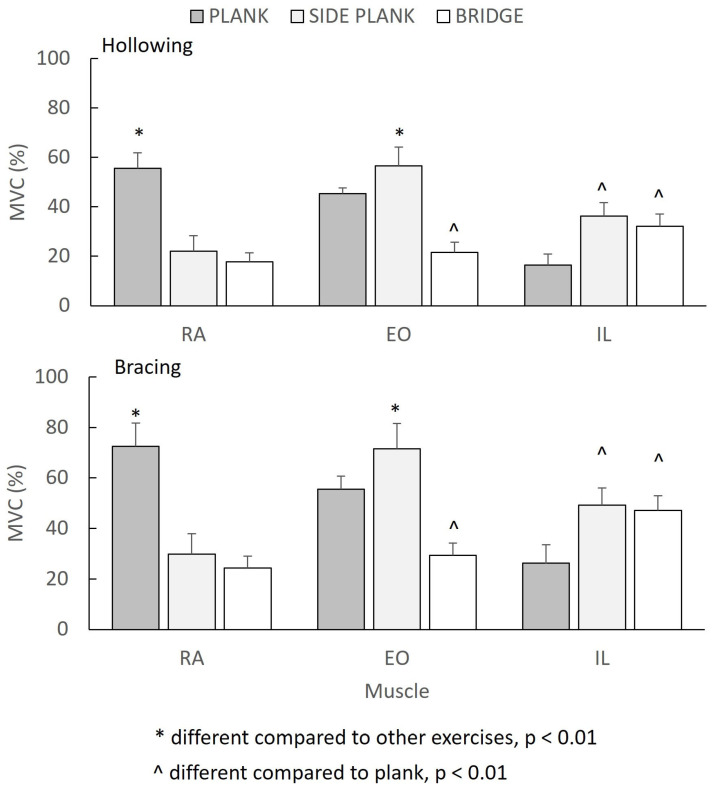
Normalized EMG muscle activation of the rectus abdominis (RA), external oblique (EO), and lumbar multifidus (LM) for each exercise and maneuver.

Figure 3 illustrates the influence of the exercise and type of maneuver on each muscle. In particular, the RA activation was greater during the plank compared to the side plank and bridge exercise (*p* < 0.01), but it was no different between the side plank and bridge (*p* > 0.05). The EO activation was greater during the side plank compared to the plank and bridge exercise (*p* < 0.01), and it was also greater during the plank compared to the bridge exercise (*p* < 0.01). Finally, IL activation was no different between the side plank and the bridge exercise (*p* > 0.05), but both of these exercises showed greater IL activation than the plank exercise (*p* < 0.01).

## 4. Discussion

The purpose of our study was to compare the effects of abdominal hollowing and bracing on local core muscle (TrA, IO, LM) thickness and global core muscle (RA, EO, IL) EMG in CrossFit^®^ trainees. The results showed that hollowing increased the thickness of the local core muscles more than bracing, while bracing caused a greater EMG activation of the global core muscles than hollowing. Owing to the three-factor interaction, different exercises showed different thicknesses or EMG for each muscle, depending on whether the individuals performed the hollowing or the bracing maneuver.

There is a general debate on whether abdominal hollowing or bracing activate different abdominal muscles [12,13]. Hence, some professionals may prefer one of these techniques over the other, without considering the validity and value of both interventions. As our results show, abdominal hollowing (across the exercises) increased the contraction thickness (which is an indirect measure of activation) of the TrA, IO, and LM more than bracing (Table 2). This agrees with some studies, which reported a much greater increase in the TrA [20,21] and IO [21] thicknesses during hollowing than bracing. However, other studies [19,22] have found different results. In particular, Kim et al. [22] found a greater TrA thickness change during hollowing than bracing, but the IO and EO thicknesses were greater in bracing than hollowing. Moghadan et al. [19] found a greater TrA thickness change during abdominal bracing compared to hollowing. The exact reason for this difference between various studies is unclear. A potential explanation may be that the exercises and testing positions differed between the studies, as some studies examined the two maneuvers from the sitting or supine position [20,21], the bridging [19], or the standing posture with sudden perturbations [22]. Another explanation may be that the instructions provided to the participants to perform each maneuver may have differed between the studies and this may have an influence on the recorded US thickness measures [19].

The results from this study showed a greater (about 20–30%) EMG activation of the global spinal muscle EMG during bracing compared to hollowing (Table 3). This is partly in agreement with some studies [16,17,18], which reported a greater RA activation during bracing than hollowing, but it contrasts with other studies which did not find differences in the RA activation between the two maneuver types [14,15]. Urquhart et al. [15] and Stanton and Kawchuk [18] found a greater EO EMG activation during bracing than hollowing, which agrees with the present findings, but Oshikawa et al. [17] found the opposite result. As already explained, differences in exercise type and instructions given to the participants may explain these conflicting findings. In addition, variations in the EMG electrode positioning and processing may also contribute to differences in the EMG findings between the studies. For example, Urquhart et al. [15] used a 3.5 cm inter-electrode distance, about 5 cm from the xiphoid process, while we used electrodes with a 1 cm inter-electrode distance which were placed on the midline between the xiphoid process and pubic symphysis. Greater inter-electrode distances may be more representative of the underlying muscle activation, but they are prone to crosstalk errors than shorter inter-electrode distances [34], while the RA architecture may vary along the muscle–tendon unit [35], thus affecting the recorded EMG patterns.

Consideration of the EMG and US findings indicate that when performing abdominal hollowing there is a greater TrA and IO thickness (Table 2), but a lower EMG global muscle activation (Table 3) compared to bracing. Nevertheless, neither the US thickness of the deep muscles during bracing nor the EMG levels of the global muscles during hollowing can be considered minimal. Utilizing EMG and US measures, Stanton and Kawchuk [18] reported that although abdominal bracing increased spinal stiffness, muscle EMG, and cross-sectional area more than hollowing, both techniques caused a significant increase in spinal stiffness relative to the standard exercise condition. This suggests that each maneuver type may serve to achieve specific training targets, but both types can enhance the activation of global and local spinal muscles. This supports Brown and McGill [27], who suggested that different contraction strategies cause very complex interactions between the abdominal wall muscles.

Within the limitations of the specific exercises examined in this study, the grading of these exercises from lower to greater recruitment varied between the muscles and it was maneuver dependent. For example, the bridge exercise with the hollowing maneuver can lead to greater TrA thickness, whereas if the bracing maneuver is performed, the side plank is preferable (Figure 1). Bridge exercises are preferable for increasing the relative thickness of the IO and LM, especially when using the hollowing maneuver. In general, the grading of the exercises varied between the superficial muscles, but it was not maneuver dependent (Figure 2). For example, the plank exercise caused a greater increase in RA activation, the side plank exercise caused a greater increase in the EO EMG, while the IL was more activated than the RA and EO in the bridge exercise (Figure 2). These results show the importance of incorporating various exercises in CrossFit^®^ workouts, to target all the muscle groups that enhance the stability and strength of the spinal muscles.

Previous research has shown that hollowing may increase lumbo–pelvic–hip complex stability [36] and pelvic floor muscle activity [37], while minimizing the facilitation of global muscular activity [36]. However, other researchers disagree that the abdominals should be hollowed out and that only the TrA and MF should be activated before dynamic exercise [8]. This is because abdominal hollowing can reduce the activation of many of the muscles that are normally active during dynamic movements, thereby preventing the natural co-contraction of other muscles that stabilize the core. Further, some studies challenge the effectiveness of treating low back pain only using abdominal hollowing [38,39]. In this way, McGill [40] understates that correct abdominal bracing should activate the TrA without the need to retract the navel and may be more valuable for lumbar spinal protection and stability than hollowing [41,42]. Hence, it has been suggested that bracing should be the focus of stabilization training [42]. Our study showed that both hollowing and bracing increased the activation of the TrA, more than the IO and LM, in all the exercises in the protocol (Table 1). However, hollowing also produced significantly greater coactivation of all three muscles during each exercise. This finding is consistent with previous research [43,44,45] that has demonstrated coactivation of the deep trunk muscles, particularly the TrA/IO. Nevertheless, abdominal hollowing is more difficult to teach and monitor than bracing. This is the reason that early studies proposed the use of real-time US imaging to teach the abdominal hollowing technique [46]. Therefore, both methods of abdominal wall activation are needed depending on the individual case.

The results from this study have important implications for CrossFit^®^ training and core stability exercises. Depending on the goal and purpose of the exercise, CrossFit^®^ trainees may choose to use either hollowing or bracing to activate their core muscles more effectively. For example, if the goal is to improve the activation of the local core muscles, and through this improve spinal stability [13,25] and prevent low back pain, hollowing may be more beneficial than bracing. This may be particularly useful when individuals display relative atrophy or weakness in the local deep muscles. In contrast, if the goal is to improve trunk strength and power, bracing may be more beneficial as it activates the global core muscles that produce trunk movements and transfer the forces between the upper and lower limbs [12,47]. Abdominal bracing can be easily taught to trainees, and it seems to be more suitable for CrossFit^®^ training, as it is a dynamic workout that involves many exercises that require load and force transfer, as well as the coactivation of multiple muscle groups. This suggestion agrees with the findings of Grenier and McGill [12] and Moghadam et al. [19], who reported that bracing coactivates the lateral abdominal and transverse muscles from a broader base, providing greater stability than hollowing.

This study has some limitations that need to be acknowledged and addressed in future research. First, this study used a small sample size of healthy adults who were skilled at CrossFit^®^ exercise. Therefore, the findings may not apply to other populations or settings, such as older adults, beginners, or patients in clinical settings. To improve the external validity and relevance of the findings, future studies should use bigger and more varied samples. Second, this study only used three exercises (plank, side plank, bridge) to measure core muscle activation during abdominal hollowing and bracing. This means the results may not reflect the effects of other exercises commonly used in CrossFit^®^, like squatting, lifting, and burpees. Future studies should use more varied and complex exercises to capture the dynamic nature and variety of CrossFit^®^ training. Additionally, this study only used US and EMG measurements to assess core muscle activation during abdominal hollowing and bracing. Therefore, the results may not provide a complete picture of core stability and performance during CrossFit^®^ exercises. Other measures or indicators of core stability and performance, such as spinal kinematics, balance tests, or functional tasks, should be used in future studies.

## 5. Conclusions

This study showed that abdominal hollowing increases the thickness of the TrA, IO, and LM more than abdominal bracing, as measured by US imaging. Abdominal bracing increases the electrical activity of the RA, EO, and IL more than abdominal hollowing, as measured by EMG. The grading of the exercises varied between the muscles and varied between the maneuvers, especially for the deeper abdominals and lumbar muscles. CrossFit^®^ practitioners can choose to use either hollowing or bracing to activate their core muscles more effectively, depending on the goal and purpose of the exercise.

## Figures and Tables

**Table 1:** Mean (±SD) anthropometric characteristics for each group. (BMI = body mass index.)

	Total Sample	Men	Women
N	24	15	9
Age (years)	29.5 ± 7.83	29.7 ± 8.63	29.3 ± 6.76
Height (cm)	173.3 ± 10.1	179.1 ± 6.9	165.1 ± 4.9
Mass (kg)	70.6 ± 12.4	76.3 ± 10.7	60.4 ± 7.5
BMI	23.3 ± 2.2	23.8 ± 2.3	22.5 ± 2.0
CrossFit years	4.2 ± 1.2	3.9 ± 2.9	5.1 ± 2.0

**Table 2:** Mean (±SD) muscle relative US thickness of the transversus abdominis (TrA), internal oblique (IO), and external oblique (EO) in each exercise using either the bracing or hollowing maneuver, expressed as a percentage of the thickness at rest.

Exercise	Bracing	Hollowing
Plank		
TrA	47.9 ± 16.0	85.9 ± 20.1 *
IO	33.3 ± 7.46 #	58.5 ± 16.7 *#
LM	15.9 ± 3.54 ^	26.8 ± 5.33 *^
Side plank		
TrA	61.2 ± 15.9	88.4 ± 11.9 *
IO	46.4 ± 8.39 #	69.2 ± 10.2 *#
LM	19.3 ± 3.71 ^	33.7 ± 4.66 *^
Bridge		
TrA	53.2 ± 8.37	94.3 ± 8.13 *
IO	50.9 ± 5.40	79.2 ± 6.72 *#
LM	38.1 ± 3.71 ^	56.9 ± 4.60 *^

* Greater than bracing, *p* < 0.01; ^ lower than IO and TrA, *p* < 0.01; # lower than TrA, *p* < 0.01.

**Table 3:** Mean (±SD) normalized EMG (expressed as a percentage of the maximum volume contraction) of the rectus abdominis (RA), external oblique (EO), and iliocostalis lumborum (IL) for each exercise and maneuver.

Exercise	Bracing	Hollowing
Plank		
RA	72.5 ± 9.17	55.6 ± 6.22 *
EO	55.6 ± 7.99 #	45.4 ± 6.41 *#
IL	26.2 ± 4.62 ^	16.4 ± 3.70 *^
Side plank		
RA	29.9 ± 5.07	22.1± 2.30 *
EO	71.6 ± 10.0 #	56.6 ± 7.65 *#
IL	49.3 ± 6.72 ^	36.3 ± 5.50 *^
Bridge		
RA	24.4 ± 7.30	17.7 ± 4.53 *
EO	29.4 ± 4.88	21.6 ± 4.18 *
IL	47.2 ± 5.69 ^	32.1 ± 5.02 *^

* Lower than bracing, *p* < 0.01; ^ different compared to the other two muscles, *p* < 0.01; # lower than RA, *p* < 0.01.

## Data Availability

The data presented in this study are available at: 10.17632/4sx58vt8v9.1 accessed on 7 August 2023.

## Supplementary Materials

The following supporting information can be downloaded at: https://www.mdpi.com/article/10.3390/sports11080159/s1, Supplementary File S1.
