# Supplementary material for: Spinal Muscle Thickness and Activation during Abdominal Hollowing and Bracing in CrossFit® Athletes
Source: Sports (Basel). 2023 Aug 18;11(8):159. doi: 10.3390/sports11080159 (PMC10458255; doi:10.3390/sports11080159)
Supplement: Supplementary file 1 [file sports-11-00159-s001.zip › sports-2537584-supplementary.pdf]

Supplementary File S1

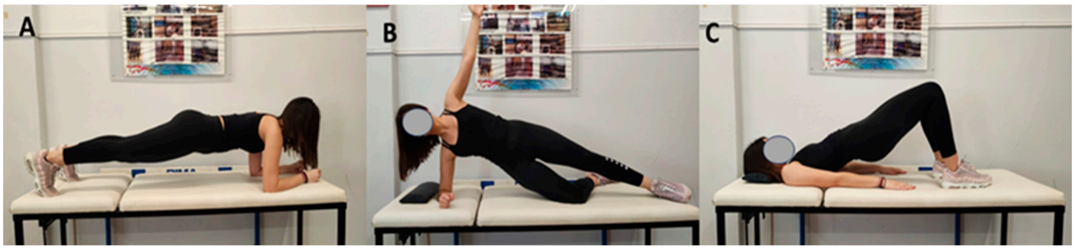

For the exercise protocol, we asked the participants to perform three classical exercises for core stability with a bracing and a hollowing of the abdomen. They were instructed to perform as follows:

A) **Plank Exercise**, "Lie face down on the floor with your forearms and toes on a mat. Your elbows should be just below your shoulders and your forearms should be facing forward. Engage your core muscles first by tightening, and then try again by drawing in and squeezing your glutes. Your body should be in a straight line from head to heels, without sagging or arching. Hold this position for as long as you have been instructed to ‘relax’.
B) **Side Plank**, "Lie on your right side with one leg straight and the other knee on the mat. Place your right elbow under your right shoulder with your forearm pointing away from you and your palm in contact with the floor. Engage your core muscles first with the tightening and then try again with the drawing in and squeezing of your glutes. Lift your hips off the mat so that your weight is on your elbow and the knee of your right foot. From your ankles to your head, your body should be in a straight line. Hold this position for as long as you have been instructed to ‘relax’.
C) **Bridge Exercise**, “Lie on your back on a mat. Bend your knees and place your feet hip width apart, flat on the floor at a 90-degree angle. Our arms should be by our side, hands down. Contract your lower back and abs as you flex your glutes and push your hips up. While holding the bridge, press your heels into the floor. Your body should form a straight line from your shoulders to your knees without sagging or arching. Try the bracing exercise first, then the hollowing exercise. Hold this position for as long as you have been instructed to “relax”.
